# Assessing the feasibility of a neck‐strength training intervention in university women's rugby

**DOI:** 10.1002/ejsc.12028

**Published:** 2024-03-18

**Authors:** Freja J. Petrie, Elisabeth M. P. Williams, Kelly A. Mackintosh, Chelsea Starbuck, Melitta A. McNarry

**Affiliations:** ^1^ Applied Sports, Technology, Exercise and Medicine Research Centre (A‐STEM) Swansea University Wales UK

**Keywords:** cervical strength, concussion, female athlete, qualitative, sports‐related brain injury

## Abstract

Cervical muscle strength has been demonstrated to reduce concussion risk in high school athletes, and interventions to improve this in male rugby players have elicited strength improvements. However, the feasibility of introducing neck‐strengthening interventions into women's rugby has not been investigated. This study sought to pilot a neck‐strength intervention in university‐level women's rugby players. A fixed‐frame dynamometer was used to assess the multi‐directional isometric neck‐strength of 14 university women's rugby players (20.3 ± 1.0 years). Between baseline and end‐of‐season testing, a neck‐strengthening program was completed. Interviews were conducted with six players and two coaches to understand program engagement. Interviews were transcribed verbatim and processed via inductive content analysis. Increases in absolute neck‐strength post‐intervention in left and right lateral flexion (left 85.4 ± 29.7 N to 108.2 ± 41.6 N, *p* = 0.02, right 87.4 ± 33.3 N to 110.5 ± 40.3 N, *p* = 0.01) and flexion (128.4 ± 28.8 N to 147.9 ± 30.5 N, *p* = 0.01) were perceived positively by the players although there were suggestions that greater adaptability according to training age and more variety was required. Participants initially demonstrated limited awareness of neck‐strength training but engaged well with the exercises once the potential benefits were understood. This intervention shows promise as an effective, palatable strategy to improve neck‐strength in university women's rugby players. Further research is needed to establish whether such improvements in neck‐strength are associated with meaningful reductions in head impact occurrence.

## Highlights

- Neck‐strength training has proved an effective strategy for reducing concussion incidence in high‐school and elite male populations but is yet to be investigated in females.
- A neck‐strengthening intervention was introduced in a team of university women's rugby players, who were later interviewed to understand their experiences of the intervention.
- Multi‐directional neck‐strength was tested via an Isometric Neck‐Strength Testing Apparatus, purpose‐built to mimic the scrum position associated with rugby.
- Improvements to neck‐strength reached significance in flexion and lateral flexion strength, and the intervention was received positively by players and coaches.

## 1 INTRODUCTION

Rugby is increasingly engaged in by women (World Rugby, 2017). Recent statistics collated by World Rugby indicate that women account for a quarter of the global rugby‐playing population (World Rugby, 2020). Within rugby, contact events are often associated with head impacts (Tierney et al., 2016), with concussion incidence in premier 15's women's rugby recorded at 12.6 per 1000 playing hours (Kemp et al., 2021). However, limited pitch‐side healthcare in the women's game may mean that concussions are underreported (Haarbauer‐Krupa et al., 2018). This is particularly concerning given that the female brain is purported to have a greater biological vulnerability to concussion (Dollé et al., 2018).

The greater vulnerability to concussion suggested in females may be attributable to sex differences in neurological structures and the coupling of cervical vertebrae that reduce the ability of females to resist inertial loading of the head or tolerate acceleration acting on the brain (Dollé et al., 2018; Stemper et al., 2008). It is also pertinent to note that, relative to body mass, the magnitude of head impacts may also be greater in females than males (Williams et al., 2021), further exacerbating the physiological predisposition.

The consequences of concussion are variable between individuals (Nelson et al., 2013). In the short‐term, symptoms may preclude normal functioning, due to, for example, leave from work, sport or education, and lead to social isolation (Graves et al., 2020; Sanderson & Cassilo, 2019). In the longer‐term, experiencing multiple concussions or repeated head impacts is associated with deficits in neurocognitive functioning (Hume et al., 2017), and the potential development of neurodegenerative disease (Nowinski et al., 2022). Therefore, strategies to minimize head‐impact events, and concussion for rugby players are urgently needed.

During a head impact, the cervical musculature can contract to increase neck stiffness (the ability of the neck to resist deformation and absorb kinetic energy) and reduce the acceleration experienced by the brain (Dezman et al., 2013; Elliott et al., 2021; Hrysomallis, 2016; Peek et al., 2022). It has therefore been suggested that the development of cervical musculature may improve the ability of the neck to stabilize the head, therefore reducing head impact magnitudes. Consequently, the role of neck‐strength (NS) training has been explored in a range of sporting populations (Collins et al., 2014; Farley et al., 2022). Specifically, in a population of mixed‐sex high‐school athletes, an increase of 0.45 kg in NS was associated with a 5% reduction in concussion risk (Collins et al., 2014). In elite male rugby, a 10% increase in Ext strength was associated with a 13% reduction in concussion risk (Farley et al., 2022). However, given the sex‐related physiological differences, whether these beneficial adaptations are similarly demonstrated in female athletes remains to be elucidated.

To quantify NS training efficacy, various devices have been used, such as dynamometers and load cells (Peek, 2022; Williams et al., 2021). To improve the ecological validity of NS testing, sports specificity, portability, NS profile and the type of muscle contraction should be considered (Peek, 2022). For rugby, NS testing in a horizontal scrum‐like position has previously been utilized (Salmon et al., 2015; Williams et al., 2021). However, there is a dearth of data pertaining to NS trainability in females.

Participant adherence is a modifiable determinant of intervention efficacy (Owoeye et al., 2018)—even if physiologically beneficial, such benefits will not be realized if the intervention is not feasible for players. In elite women's sport, facilitators to engagement with injury prevention (IP) programs include positive attitudes to IP strategies across stakeholders (teammates and coaches) and coach supervision to assess form and feedback on progress (Bruder et al., 2021). Concomitant barriers to intervention uptake included insufficient facilities, time pressure and lack of understanding about how the intervention could be beneficial (Bruder et al., 2021). Consideration of player experiences is therefore vital in the assessment of an intervention's efficacy, but little is known regarding player perceptions of NS training.

Therefore, the aim of this pilot study was to implement a NS intervention in a university women's rugby team and to ascertain participant's perceptions of the training intervention.

## 2 METHODS

### 2.1 Sample

A British university women's rugby union team was invited to participate. Initially, 19 players provided written informed consent to take part, however, five players were excluded from the study due to injury or educational commitments. A total of 14 participants completed the entire investigation. Ethics approval was granted by the Swansea University Research Ethics Committee (application number FP_01‐10‐21) and the study was conducted in accord with the Declaration of Helsinki.

### 2.2 Research tools

Isometric NS was tested via an Isometric Neck‐Strength Testing Apparatus (INSTA) as detailed elsewhere (Williams et al., 2021). The INSTA measured strength in Newtons (N) via fixed‐frame dynamometry whilst the participant was in a rugby‐specific prone position. The participant's test position was standardized as per Williams et al. (2021), with strapping around the torso and legs to limit recruitment to the cervical musculature.

### 2.3 Neck‐strength testing protocols

NS was tested at a pre‐season familiarization session (week 1), at baseline (week 2) and at the end of the season (22 weeks later). In the familiarization session, player anthropometrics (height, body mass and head and neck circumference) were measured in accordance with the International Society for the Advancement of Kinanthropometry guidelines (Norton, 2018). The familiarization session was used to reduce any learning effect that may occur as participants were introduced to new exercises (Burnstein et al., 2011; Haider et al., 2020). Testing procedures are detailed in Table 1.

**TABLE 1:** Testing procedures.

Activity	Procedure
Warm‐up prior to INSTA use	Participants completed 10 arm circles forwards, then backwards, followed by 10 press‐ups supported by the knees.
	Participants then completed three repetitions of three second isometric head lifts in each direction.
Isometric head lifts	Participants lay supine and were instructed to tuck their chin to their chest before lifting their head three inches off the floor for 3 seconds, before returning their head to the starting position. These lifts were completed three times in supine and prone positioning and from a left and right side‐plank. For the side plank, participants were instructed to keep their hip on the floor so that difficulties balancing did not detract from warming up the neck.
Familiarization testing	Warm‐up.
	50% trial: Participants were then asked to push against each of the four load cells (flexion, extension, left lateral flexion and right lateral flexion) at 50% of their perceived maximal effort.
	70% trial: Participants were then asked to complete a maximum voluntary contraction at 70% perceived maximal effort for 3 seconds. Between the 50% and 70% conditions the participant rested for 30 seconds.
Maximum voluntary contraction testing	Warm‐up.
	50% trial (as above).
	100% trial (identical to the 70% trial, but with maximum voluntary contraction).

NS testing took place 1‐week post familiarization, and not within 2 days prior to or post matches. Participants rested for 30 s between 50% and 70%, and 50% and 100% trials respectively. The order of testing was randomized across directions between participants.

### 2.4 Neck‐strength training protocols

The NS training program began with bodyweight isometric holds, then progressed to banded isometric holds, as used successfully in Becker et al. (2019). NS exercises were incorporated into established bi‐weekly strength and conditioning sessions and were performed at the end of the session as a team. To achieve progressive overload, the duration of isometric holds, repetitions or band strengths were increased each week (Table 2).

**TABLE 2:** Neck‐strength program.

Timeline (weeks)	Program details
1–3	Head lifts with body weight only. Repetitions of isometric head lifts progressed each week to 3, 4 and 5 sets of 5 s.
4–6	Light (approximately 1 mm thick) resistance bands were introduced. Players worked in pairs or tied the bands to a squat rack and completed the isometric holds, in each direction, on their knees in an upright position. Holds progressed from 3 to 4 × 5 s.
7–12	Field training, neck‐strengthening, and matches ceased for 5 weeks during university holidays and examinations. Players were given a personalized strength and conditioning program to follow at home.
13–18	Matches, field training and neck‐strengthening recommenced. A medium resistance band was used (approximately 2 mm) used and 3, 4, 5 × 5 s holds completed.
18–22	A heavy resistance band was used (3 mm thick), and isometric holds were sustained for 3, 4 then 5 sets of 8 s.

Adherence to the program and correct form was encouraged at each session by the coaches and by the researcher.

### 2.5 Semi‐structured interview protocols

Post‐intervention, participants and coaches were invited to participate in a semi‐structured online interview about their experience of the NS testing and training. The purpose of these interviews was to understand how players experienced the testing sessions and intervention, and obtain feedback regarding engagement and feasibility that could be used in the design and refinement of interventions. Interviews were conducted by the lead author, a former women's rugby player, and structured in accordance with the interview guide (Supporting Information S1). Ten participants and two coaches were invited for an interview, of which three forward and three back players and both coaches agreed to be interviewed. Player and coach interviews lasted approximately 17 and 27 min respectively and were transcribed verbatim.

Content analysis, which refers to describing and interpreting qualitative data and placing data into categories and themes via a systematic coding process (Assarroudi et al., 2018; Elo & Kyngäs, 2008), was conducted using NVivo 12 (NVivo qualitative data analysis Software; QSR International Pty Ltd. Version 12, 2019) for Windows. Given the relative novelty of the intervention, an inductive approach to analysis was deemed most appropriate. Interview data was therefore coded twice; initially to identify key patterns and themes, then subsequently to confirm allocation to these codes (Graneheim et al., 2017; Krippendorff, 2019).

### 2.6 Statistical analyses

Quantitative data were analyzed using SPSS 28.0 (IBM Statistics ver. 28.0). for Windows. The normality of data and population variances were explored using Shapiro‐Wilk and Levene's tests, respectively. Subsequently, parametric and non‐parametric tests were used as appropriate. Independent and paired *t*‐tests were used to analyze anthropometric data and pre‐post intervention strength changes, respectively. For baseline and post‐intervention data, descriptive statistics and positional variations were calculated and peak values identified for neck flexion (Flx), extension (Ext), left lateral flexion (Llf) and right lateral flexion (Rlf). NS imbalances were calculated between Flx and Ext, and left and right lateral flexion. Relative strength (Newtons per kg of body mass) was also calculated for each direction.

## 3 RESULTS

Within this section, quantitative and qualitative results are combined to understand the feasibility of the intervention. Overall, eight qualitative sub‐codes were identified, which were grouped into three main codes, namely awareness, training program and testing format (Table 3).

**TABLE 3:** Codes identified within interview transcripts.

Code	Sub‐codes
Awareness	Reasons for awareness
	Value of awareness
Training program	Intervention format
	Suitability for various training ages
	Equipment
	Coach presence
Testing format	Participant comfort
	Testing frequency

### 3.1 Anthropometrics

Whilst there were no significant positional differences in height, head circumference or age, forwards demonstrated a greater body mass (82.0 ± 14.9 vs. 66.7 ± 7.8 kg; *t* (11) = 2.1, *p* = 0.03) and neck circumference (33.3 ± 1.2 vs. 31.9 ± 1.4 cm; *t* (11) = 1.8, *p* = 0.05) than backs. There was no significant positional difference in terms of the ratio of head‐to‐neck circumference (1:1.7 IQR 0.1 cm). Overall, participants were 20.3 ± 1.0 years, 169.1 ± 7.2 cm tall, weighed 76.1 ± 13.9 kg, and had a head and neck circumference of 55.2 ± 1.9 cm and 32.8 ± 1.4 cm, respectively.

### 3.2 Baseline neck‐strength

As this was the first time most players had completed NS testing, players were asked about their perceptions of the equipment and testing methods. Players mentioned the design of INSTA seemed *“robotic”* and unusual: *“I think first impressions of it [pulls face] if you haven't done it before*, *looks quite daunting”* (Ps3 club back). However, players did note that the INSTA became less daunting through use: “*some people found it a bit scary going into the rig [INSTA]*. *Then*, *once you've done it once*, *it was fine”* (Ps5). Testing duration and positioning of the INSTA during use were also highlighted as factors that affected participant comfort: *“it was a bit weird getting strapped into it like*, *I think if you're claustrophobic it's not very good*. *But it was a quick process*. *And like*, *it would encourage people to go back to it”* (Ps5 club back). However, this stress was reduced by testing alongside teammates: *“definitely doing them in groups takes off the stress off it”* (Ps5 club back).

At baseline, absolute measures of NS did not significantly differ by positional grouping, however, forwards had significantly greater relative strength in Llf; *t* (11) = −2.05, *p* = 0.03, *g* = 1.09) and Flx; *t* (11) = −2.05, *p* = 0.03, *g* = 1.08). There was a trend for positional differences in Ext; *t* (11) = −1.64, *p* = 0.06, *g* = 0.87; Table 4). At baseline, there were no significant differences in percentage imbalances between Flx and Ext (14.4 ± 2.6%) or between Llf and Rlf (12.3 ± 6.9%).

**TABLE 4:** Neck‐strength results.

	Baseline absolute strength (N)	Post‐intervention absolute strength (N)	Post‐intervention percentage increase (%)	Baseline relative strength (N⋅kg^−1^)	Post‐intervention relative strength (N⋅kg^−1^)
Left lateral flexion	85.4 ± 29.7	108.2 ± 41.6	26.7	1.1 ± 0.4	1.4 ± 0.5
Right lateral flexion	87.4 ± 33.3	110.5 ± 40.3	26.4	1.2 ± 0.5	1.5 ± 0.6
Flexion	128.4 ± 28.8	147.9 ± 30.5	15.2	1.6 ± 1.3	2.0 ± 0.4
Extension	131.3 ± 32.3	140.5 ± 24.6	9.2	1.8 ± 0.5	1.9 ± 0.4

### 3.3 Neck‐strength training intervention

Interviewed players and coaches reported that they would prefer NS training to take place at the start of a training session, rather than towards the end, due to fatigue. Players preferred completing the exercises as a group, stating that *“it was quite light‐hearted*, *and that made people enjoy doing it* (Ps4 club forward)” and *“it made people actually work harder* (Ps3 club back)”. A player that had completed NS exercises outside of the team strength and conditioning sessions noted a different effect when training alone, as she felt less able to isolate her neck when the resistance bands were tied to a squat rack compared to when a partner was assisting. Indeed, the value of the coaches providing encouragement and support during the training session was highlighted by players, who perceived that the intervention would be less effective without the input of the coach:

I think that's important for the coaches to stay at a training session, just so that we don’t forget the exercises. The coach does influence how well we do in a training session. So, if he's not happy with how we're performing, he will tell us. Yeah, that's really important.

(Ps6 club forward).

One club player suggested that the lack of a controlled environment in typical club‐level rugby may hinder adherence to the NS program:

Because at the high‐performance level, you have all these gym sessions that are scheduled in. I think it's translating that into a club environment when you're very much on your own. So, you haven't got all this special support like a strength conditioning coach, I think it would be harder to enforce that at a club level.

(Ps2 club back).

The first stage of the intervention consisted of bodyweight head lifts, however, certain players expressed that they felt this initial stage was unnecessary. Further, an ex‐national team player (Ps1) noted that rugby itself may act as a training stimulus, progressing NS beyond a point where bodyweight holds could improve strength: “*I've had my six years of experience already*. *I think I've already established a certain NS for that position*. *So without the resistance bands*, *I didn't find it challenging”*. In contrast to these concerns regarding overload and progression, absolute NS significantly increased from baseline to post‐intervention in Llf (*t* (12) = −3.51, *p* = 0.02), Rlf (*t* (12) = −2.92, *p* = 0.01), and Flx (*t* (12) = −3.46, *p* = 0.01), whilst increases in Ext approached significance (*t* (12) = −1.69, *p* = 0.06). These changes were associated with large effect sizes for Llf (*g* = 0.94), Rlf (*g* = 0.78) and Flx (*g* = 0.93) and moderate effect sizes for Ext (*g* = 0.46). Post‐intervention, NS did not significantly differ by positional grouping. Relative strength increased in all directions (Llf (*t* (12) = −3.67, *p* < 0.01), Rlf (*t* (12) = −3.12, *p* < 0.01), Flx (*t* (12) = −3.25, *p* < 0.01)), except for Ext. Post‐intervention, there were no significant imbalances between Flx and Ext (14.6 ± 8.1%), or Llf and Rlf (16.6 ± 11.1%) by position. Furthermore, directional imbalances showed no significant change between baseline and post‐intervention.

Both the coaches and players reported that they would like NS training to be included at the start of a field training warm‐up: “*if you incorporated 5 minutes into the start of a field session*, *then you'd feel a bit more warmed‐up and ready for contact”* (Ps4 club forward). Whilst the resistance bands were praized due to their portability, the strength and conditioning coach highlighted that a progression from resistance bands would likely be needed for future training and noted that a weighted‐pulley system would be *“ideal”*.

Participants were asked about the likelihood of performing NS training exercises outside of strength and conditioning sessions, either at home or in a public gym. Players that were unlikely to complete NS training exercises at a gym stated that they had their set routines and perceived that NS training outside of the season would have little value. However, these players were open to changing this view:

I do gym *[outside of team provided sessions]* and I would not think about it *[NS training]* because I've got my own routine. I guess I'd have to incorporate it in. But I wouldn't see any use in doing it. I guess it's to keep like, your NS up for next season. I kind of look at it as a warm‐up for your neck. So, if I'm gonna do that before my gym session, it would be useless.

(Ps1ex‐national level forward).

Players who had completed NS training exercises in public gyms noted that, whilst the exercises may be unconventional, they would still feel comfortable enough to complete them: *“I look quite funny doing them*, *but I wouldn't be too worried about doing them in a public gym”* (Ps4 club forward); and *“I do feel stupid*. *But … I know it's important*. *So*, *then you would do it”* (Ps6 club forward).

Players reported that they would prefer more frequent testing to understand how their NS was progressing: “*more regular testing*, *it'll just give you that bit of confidence that it is actually working*” (Ps1 ex‐national team forward). The idea of more frequent testing was also recommended by the strength and conditioning coach, who suggested that testing every 3 months would align with his programming schedule, but also enable players to see any changes.

Players felt that the NS training was valuable, often referencing the high incidence of concussion in rugby: *“especially when you're playing a sport like rugby where concussion is so high*. *Yeah*, *I think it's* [implementing head impact reduction strategies] *important”* (Ps1 ex‐national forward),; “*it's* [concussion] *such a big problem*, *… I think that something definitely needs to be in place”* (Ps6 club forward).

Players highlighted the importance of education for engagement to prescribed NS training programs, and would welcome greater information on NS training:

I would find it *[learning more about injury prevention strategies]* really interesting. And then it motivates me to do more of it, when you know the benefits of it, rather than “Oh, I've got to do that. I don't know what it's doing to me.”

(Ps1 ex‐national forward).

Both coaches concurred with this, and the field coach suggested that greater understanding about the purpose of the exercises would further engage players:

What I’ve found when coaching them is that if they don't understand how something is going to help them, they're not going to really trust it… But once they do understand it and they think the drill is good, then they'll do it

(field coach).

Of those interviewed, only an ex‐national team player had previously been aware of NS training, noting she was also studying a Sports Science degree, and enjoyed learning about IP. When players were asked if they had been aware of NS training prior to being involved in the intervention, they generally reported that the potential benefits of NS training were not public knowledge. However, players expressed that there would likely be an interest in NS research should the rugby community become aware of its benefits:

Most people I play with in club are farmers or mechanics and stuff. They don’t have any knowledge about muscles and NS or anything. So I think *[that]* if it was introduced to them, I think people would be interested in that.

(Ps2 regional back player).

Limited familiarity with sports science and access to qualified coaches acted as a barrier to exposure to NS training: *“I played for quite a small club*, *so it was kind of people's dads coaching*. *It was just play but nothing else*” (Ps4 club forward player).

Neither the strength and conditioning, nor field coach had been aware of NS training as an IP strategy, despite both coaches having considerable experience coaching and playing rugby. Rather, NS had only been considered in performance contexts, particularly in preparing players for scrummaging, using self‐applied manual resistance. The field coach highlighted difficulties accessing new research as the reasoning behind the limited awareness of NS training:

I’ve never really got data on anything from *[other coaches]* or anyone. In terms of how I coach, I'm constantly looking at new drills and try and look at new things online. I was never like, drip‐fed anything about NS or how to coach that.

## 4 DISCUSSION

This pilot study sought to investigate the feasibility of introducing a neck‐strengthening intervention to university‐level women's rugby players and understanding stakeholder perceptions of the intervention. Whilst this is a pilot study and mid‐season injuries reduced the sample size, the effect sizes associated with this intervention were encouraging. Insufficient female data is currently available to ascertain the significance of these differences regarding the smallest worthwhile difference in NS that translates to meaningful reductions in head impact occurrence. In males, the relationship between NS and concussion incidence has been quantified, with a 10% increase in Ext strength associated with a 13% reduction in concussion incidence (Farley et al., 2022). Further research is warranted to establish such thresholds in females and understand how such interventions may be introduced effectively into women's rugby teams.

Baseline NS in the current study was less than reported from other populations of female university rugby players (Williams et al., 2021), in which Flx and Ext were approximately 12% greater, and lateral flexions 31% greater than reported in this study. These inter‐study disparities may be explained by the differing experience of players in each study, with the population from Williams et al. (2021) playing within a higher league. As expected, previously reported strength values measured from male players (using an identical methodology to the current study) were considerably greater, with males from Williams et al. (2021) population being 111.1%, 86.4% and 131.0% stronger in Flx, Ext and lateral flexions, respectively. Potentially, the impact of the COVID‐19 pandemic on game and training time may have contributed to the lower baseline values observed in the current study. Such variation in NS between sexes highlights the potentially deleterious consequences of extrapolating male data to female athletes. Indeed, training age and baseline strength must be considered and will likely influence intervention engagement and outcomes.

Post intervention, NS values increased by 9.2%–26.7%, depending on direction. The limited research in women's rugby reduces opportunities for population specific comparisons, however, an implementation of a band‐resisted NS training program in an elite men's rugby team elicited changes of 17.4%, 12.8%, 35.0% and 7.7% in forwards and 6.3%, 2.5%, 9.3% and 5.0% in backs, in Llf, Rlf, Flx and Ext, respectively (Gillies et al., 2022). The variation in percentage change between positional groupings was attributed to forward players beginning the intervention earlier, however, in both the current study and Gillies et al. (2022), Ext experienced the least change between baseline and post‐intervention measures. Further research is needed to understand the training effects on differing muscle groups to improve the intervention efficacy.

Players from the current study reported a lack of awareness of NS training, and the coaches had only been informed of neck‐strengthening in the context of improving scrum performance. However, in surveys of stakeholder responsibility to minimize concussion in community rugby, NS training being a viable intervention was cited by medics and some coaches (Clacy et al., 2015). The presence of this knowledge in research communities, but less so in coaches or players suggests that improvements in knowledge transfer are required. In high performance coaches (from a range of sports), poor format of information and limited potential for practical applications acted as a barrier to successful knowledge transfer (Nkala & Coopoo, 2022). Furthermore, a gap was identified between the knowledge offered to coaches by sport scientists, and the knowledge that coaches would like (Nkala & Coopoo, 2022). The variation in awareness of NS training highlights a need for greater collaboration between researchers and stakeholders, particularly considering that players and coaches have demonstrated interest in this area and welcomed opportunities for further education.

Players and coaches expressed a preference for neck‐strengthening to be completed at the start of a session and as a group. The enjoyment of group‐implemented strength training has previously been expressed by women's elite Australian Football, who agreed that completing IP exercises as a team was *“a lot more fun”* and could improve motivation (Bruder et al., 2021). Completing exercises as a group may also improve adherence to the prescribed exercises, as athletes can be more easily monitored by the coach. In the current study, this was supported as players believed that they would work harder when training as a group. The reported preference for group IP training may mitigate potential loss of engagement in environments where adherence monitoring is not possible, as players are less likely to forget the exercises or become disengaged.

Whilst valuable in providing insights that may aid the design of future interventions, the results of this study should be interpreted with caution. Indeed, further research with a larger sample size is warranted, as well as in a variety of women's rugby environments to understand demographic barriers to, and facilitators of, NS training. It is also pertinent to note the dynamic nature of rugby, which isometric strength measures arguably fail to represent. However, the purpose of this intervention was to increase the ability of the player to resist acceleration of the head and maintain neutral head positioning. Therefore, the measurement of isometric values was deemed most appropriate, whilst simultaneously being the safest form of measurement (Peek, 2022).

The current bi‐weekly NS intervention was associated with encouraging improvements in NS with minimal equipment and time‐efficient exercises. It therefore has the potential to be an engaging, effective intervention that may result in improvements in NS that translate to meaningful reductions in head impact exposure.

## CONFLICT OF INTEREST STATEMENT

The authors declare no conflict of Interest.

## Supporting information

Supporting Information S1
