# Supplementary material for: Assessing the feasibility of a neck‐strength training intervention in university women's rugby
Source: Eur J Sport Sci. 2024 Mar 18;24(4):466–73. doi: 10.1002/ejsc.12028 (PMC11236057; doi:10.1002/ejsc.12028)
Supplement: Supplementary file 1 — Supporting Information S1 [file EJSC-24-466-s001.pdf]

Supplementary file 1

Interview guide for players

1. Participant consent will be obtained prior to the start of the interview, and the interviewer will introduce themselves and explain the purpose of the interview

We are conducting these interviews to understand how you felt about the neck strength training sessions and the neck strength testing. We would like your feedback, good or bad so that we can adapt the training and testing to best suit your needs. Just a reminder that anything you say will be anonymised in any publications, and you are more than welcome to stop the interview at any time.

2. The interview then begins by asking the participants:

-Please could you tell me about your journey into rugby?

The interviewer will take notes on demographics or specific experiences that may be relevant for follow-up questions later in the interview.

3. Had you ever heard of neck strength training prior to this study?

- specifically in rugby or in other sports?
- where did you hear about neck strength training?

4. When the neck strength training was first introduced to the team, what did you think of it?

- how did it fit into your normal training?
- did you like how the training was delivered (as a group, in strength and conditioning training sessions)?
- did you feel the training was beneficial?
- in an ideal world, how would you like the training to be?
- is there anything you would like to change about the neck strength training sessions, why is that?
- did the coaches affect how you trained?
- would you feel comfortable and/or confident doing these exercises in a public gym?

5. Have you ever had your neck strength tested before?

-what did you think when you first saw the INSTA?
-was testing a comfortable experience for you?
- are there any changes you would like to the testing in terms of methods, or how often we tested you?

6. Is there anything else you would like to mention?

The interviewer will then wrap up the interview, thank the participant and ensure that the participant has the researchers contact details should they have any further questions.

Interview Guide for Coaches

1. The interview will begin as described for the player interviewees
2. The interview then begins by asking the participants:

   -Please could you tell me about your journey into rugby coaching?

   The interviewer will take notes on demographics or specific experiences that may be relevant for follow-up questions later in the interview.

3. Had you ever heard of neck strength training?

   - specifically in rugby or in other sports?
   - where did you hear about neck strength training?

4. When the neck strength training was first introduced to the team, what did you think of it?

   - how did it fit into their normal training?
   - did you like how the training was delivered (as a group, in strength and conditioning training sessions)?
   - did you feel the training was beneficial?
   - in an ideal world, how would you like the training to be?
   - is there anything you would like to change about the neck strength training sessions, why is that?
   - how do you think the players engaged with the program?
   - now that you are aware of neck strength training, do you think its introduction into the team was valuable?

5. Have you ever had yours, or your player's neck strength tested before?

   -what did you think when you first saw the INSTA?
   -did you receive any feedback from the players about neck strength testing?
   - are there any changes you would like to the testing in terms of methods, or how often we tested players?

6. Is there anything else you would like to mention?

The interviewer will then wrap up the interview, thank the participant and ensure that the participant has the researchers contact details should they have any further questions.
